# Elevated *Aspergillus*-specific antibody levels among HIV infected Ugandans with pulmonary tuberculosis

**DOI:** 10.1186/s12890-017-0500-9

**Published:** 2017-11-21

**Authors:** Richard Kwizera, Rosalind Parkes-Ratanshi, Iain D. Page, Christine Sekaggya-Wiltshire, Joseph Musaazi, Jan Fehr, Barbara Castelnuovo, Andrew Kambugu, David W. Denning

**Affiliations:** 1 Infectious Diseases Institute, College of Health Sciences, Makerere University, P.O.BOX 22418 Kampala, Uganda; 2 Cambridge Institute of Public Health, University of Cambridge, Cambridge, UK; 3 The University of Manchester, Manchester, UK; 4 National Aspergillosis Centre, University Hospital of South Manchester, Manchester, UK; 5 Infectious Diseases and Hospital Hygiene, University Hospital, Zurich, Switzerland

**Keywords:** Aspergillosis, HIV, Tuberculosis, Fungal diagnostics; serology

## Abstract

**Background:**

The incidence of tuberculosis (TB) is high among human immunodeficiency virus (HIV) infected Ugandans. Recent evidence suggests that Chronic Pulmonary Aspergillosis and *Aspergillus* sensitisation might be responsible for significant mortality in patients treated for tuberculosis in Uganda.

**Methods:**

We retrieved and tested paired serum aliquots for 101 HIV-TB co-infected patients at the beginning and week 24 of TB treatment. We tested samples for *Aspergillus*-specific immunoglobulin G (IgG) and immunoglobulin E (IgE) using ImmunoCAP®; and *Aspergillus*-specific IgG and total serum IgE using Immulite® immunoassays. We compared antibody levels between baseline and week 24, relating them to selected baseline characteristics.

**Results:**

10% of the patients had elevated *Aspergillus*-specific IgE (*Aspergillus* sensitization) and *Aspergillus*-specific IgG antibodies were elevated in 9% of the patients at the end of TB treatment. There was a significant fall in the *Aspergillus*-specific IgG antibody levels between baseline and week 24 (*P* = 0.02). Patients with cluster of differentiation 4 (CD4) T-cell count <100 cells/μl and those who were not on anti-retroviral therapy at baseline had more elevated *Aspergillus*-specific IgG antibodies (*P* = 0.01, *P* = 0.03). The ImmunoCAP® *Aspergillus*-specific IgG antibody titres were higher at week 24 than baseline with more positives at week 24; even though the difference in means was small. However, this difference was statistically significant (P = 0.02). Pulmonary infiltrates were the commonest x-ray abnormality and only 5% of the patients had pulmonary cavities on chest x-ray at week 24.

**Conclusion:**

These results suggest that *Aspergillus* infection may complicate active pulmonary TB and further studies including fungal culture and thoracic imaging may now be indicated to measure the prevalence of pulmonary aspergillosis complicating tuberculosis.

**Trial registration:**

The SOUTH trial was registered prospectively. ClinicalTrials.gov Identifier: NCT01782950; Registration date: 4th February 2013; Last verified: 13th April 2015.

**Electronic supplementary material:**

The online version of this article (10.1186/s12890-017-0500-9) contains supplementary material, which is available to authorized users.

## Background

Tuberculosis (TB) remains one of the major causes of morbidity and mortality worldwide with the highest burden found in Africa and Asia, mainly linked to the human immunodeficiency virus (HIV) epidemic [1]. The 2016 World Health Organization (WHO) report on TB revealed that there were an estimated 10 million new cases of TB in the year 2015 worldwide, with 11% having HIV. An estimated 1.8 million people died due to TB in 2015, including 0.4 million deaths due to HIV/TB co-infection. The incidence of TB among HIV patients in Uganda was estimated at 202 new cases per 100,000 population [2].

Due to the limited published data on fungal disease epidemiology in sub-Saharan Africa, a recent review attempted to estimate the burden of fungal infections in Uganda using specific populations [3]. In this review, chronic pulmonary aspergillosis (CPA) was estimated at 12–22% in TB patients with cavities and 1–4% in those without cavities. Considering post-TB data in Uganda, asymptomatic CPA was estimated at 7% with an additional 1.7% having detectable *Aspergillus*-specific immunoglobulin G (IgG) antibodies with cavitation.

Recent work done in Northern Uganda has validated some of these estimates, with a CPA prevalence of 8.2%, and 6.7% having cavities among patients who had been successfully treated for pulmonary TB within the last 7 years [4, 5]. More results from this work showed *Aspergillus*-specific IgG antibody levels were raised in 26% of patients with “smear negative TB” and suggested that previously unrecognized CPA might be responsible for significant mortality in patients treated for TB in Uganda [6–8]. Beyond this limited data, little is known about the epidemiology of fungal colonisation and sensitisation, and their contribution to TB disease progress and treatment outcomes in Uganda where pulmonary TB is very common, in part driven by the high prevalence of HIV [9].

We hypothesized that patients with pulmonary TB may get colonized with *Aspergillus* during and in the post treatment period leading to a chronic lung infection and/or allergic fungal disease if the patient was pre-sensitized to *Aspergillus* antigens. Pulmonary cavitation is a pre-disposing factor for CPA [10] and may or may not present together with *Aspergillus* sensitization. We therefore aimed to establish and compare *Aspergillus*-specific antibody levels among HIV-infected Ugandans with TB, at the beginning and end of TB treatment; using ImmunoCAP® and Immulite® immunoassays.

## Methods

### Study design and population

This was a nested cohort study under the “Study on Outcomes related to Tuberculosis and HIV drug concentrations in Uganda” (SOUTH) (ClinicalTrials.gov: NCT01782950). SOUTH was a prospective study investigating the correlation of anti-tuberculosis drug concentrations and TB treatment outcomes in HIV-infected individuals with pulmonary TB at the Infectious Diseases Institute, Kampala, Uganda [11]. All participants were HIV-TB co-infected patients above 18 years with a diagnosis of their first episode of pulmonary TB i.e. proven or highly suspected TB considered for TB treatment qualifying for 6 months (24 weeks) anti-TB drugs regimen (2 months HRZE [isoniazid (H), rifampicin (R), pyrazinamide (Z) and ethambutol (E)] and 4 months HR [isoniazid (H) and rifampicin (R)]). Patients with multi-drug resistant TB were excluded. Only 32% of the participants already had an HIV diagnosis with an average time since diagnosis of 53 days. The rest of the participants were diagnosed at TB screening. Each participant was followed up for 24 weeks.

### Study samples

Samples used for this study were stored serum samples from the SOUTH study population (stored at −80°c). We cross checked the sample storage inventories to look out for participants that had a sample stored at beginning (baseline) and end of TB treatment (week 24). We included all patients with two aliquots i.e. one at baseline and one at week 24. Samples were then shipped and tested at the Mycology Reference Centre Manchester and Christie Hospital, Manchester, United Kingdom (UK).

### Study assays

Testing was initially done using the ImmunoCAP® machine (ThermoFisher®, previously Phadia) to check for levels of *Aspergillus*-specific IgG (Asp IgG) at baseline and week 24. Also, using ImmunoCAP *Aspergillus*-specific IgE (Asp IgE) antibody levels were checked at week 24 only. Further testing for total serum IgE (TIgE) levels at both baseline and week 24 was undertaken on the Immulite 2000® machine (Siemens). Remaining serum was re-tested for *Aspergillus*-specific IgG levels using the Immulite machine (Additional file 1).

### Assay cut-offs

We used a cut-off of 40 mg/l for the ImmunoCAP *Aspergillus*-specific IgG levels as recommended by manufacturer, and a cut-off of 20 mg/l for the Immulite *Aspergillus*-specific IgG levels based on preliminary results of a cross-sectional survey in Uganda [4]. Variations in the cut-off for total serum IgE have been reported ranging from 150 to 1000 UI/ml [12]. However, a cut-off of 1000 UI/ml is recommended for diagnosis of allergic bronchopulmonary aspergillosis (ABPA) [13]. Therefore, for our study, we used a cut-off of 170 UI/ml to check for general allergy and a cut-off of 1000 UI/ml as a screen for evidence of ABPA. A cut-off of 0.35 kU/l was used for *Aspergillus*-specific IgE levels as recommended by the manufacturer.

### Data analysis

Data were analyzed using STATA® version 13 (STATA, College Station, Texas). Our primary data analysis aimed at comparing the *Aspergillus*-specific IgG antibody levels at baseline compared with week 24, and relating them to baseline demographics and clinical parameters (Additional file 1) at a 95% confidence interval.

## Results

### Study population characteristics

Of 268 patients in the SOUTH study, we selected all participants (*n* = 101) with a pre and post treatment serum sample; that were enrolled into the SOUTH study between March 2013 and July 2014. Of participants, 56% (56/101) were men and the median age of all participants at TB diagnosis was 33 years (interquartile range (IQR), 27–38). All participants were HIV-infected adults with a median baseline CD4 T cell count of 155 cells/μl (IQR, 31–269, *n* = 98). Only 31% (31/101) of the participants were receiving anti-retroviral therapy (ART) at TB diagnosis. At baseline, 41% (41/101) had chest pain, 27% (27/101) had difficulty in breathing, 94% (95/101) had a cough and 89% (90/101) had sputum production with a median cough duration of 4 weeks (IQR, 3–12, *n* = 95) (Table 1).

**Table 1:** Characteristics of the study population at TB diagnosis

Descriptions	N	Statistics
Baseline Demographics		
Males, n (%)	101	56 (56)
Age at TB diagnosis, mean (SD)/ median (IQR)	101	34 (8)/ 33 (27, 38)
On ART at TB diagnosis, n (%)	101	31 (31)
CD4 (cells/μL) at TB diagnosis, median (IQR)^a^	98	155 (31, 269)
Respiratory symptoms at TB diagnosis		
Cough, n (%)	101	95 (94)
Cough duration in weeks, median (IQR)^a^	95	4 (3, 12)
Chest pain, n (%)	101	41 (41)
Difficulty in breathing, n (%)	101	27 (27)
TB diagnostics		
Positive Sputum smear, n (%)^a^	97	46 (47)
Abnormal Chest X-ray, n (%)^a^	88	80 (91)
Pulmonary Infiltrates on chest x-ray, n (%)^a^	80	73 (91)
Pleural Effusion on chest x-ray, n (%)^a^	80	15 (19)
Cavities on chest x-ray, n (%)^a^	80	5 (6)
Positive Genexpert, n (%)^a^	33	24 (73)
Positive MGIT, n(%)^a^	94	64 (68)
Positive LJ culture, n(%)^a^	92	55 (60)

Data presented are percentages (%), standard deviations (SD) and interquartile ranges (IQR)

*EH* ethambutol and Isoniazid, *RH* rifampicin and Isoniazid, *MGIT* Mycobacteria growth indicator tube, *LJ* Löwenstein–Jensen

^a^Some parameters have *N* < 101 due to missing data

TB was diagnosed by direct microscopy (smear), genexpert-MTB/RIF®, Mycobacteria growth indicator tube (MGIT) and Löwenstein–Jensen (LJ) culture. Only 47% (46/97) of the participants had positive sputum smears at TB diagnosis and 73% (24/33) had positive sputum genexpert MTB/RIF results. 68% (64/94) of the participants had positive MGIT while 60% (55/92) had positive LJ cultures. For participants who had chest x-rays done at baseline, 91% (80/88) had abnormal x-rays. The commonest chest x-ray abnormalities registered were pulmonary infiltrates (91% [73/80]) followed by pleural effusion (19% [15/80]) and cavities (6% [5/80]) (Table 1).

### Aspergillus-specific IgG antibody levels using ImmunoCAP®

Using the ImmunoCAP immunoassay, the baseline median *Aspergillus*-specific IgG antibody levels were 4.43 mg/l (IQR, 2.07–7.17) with 4% (3/76) of the patients having elevated levels (>40 mg/l); while at week 24, the median *Aspergillus*-specific IgG antibody levels were 4.9 mg/l (IQR, 2.13–9.14) with 2% (2/94) having elevated levels (Table 2). All three patients who were positive for *Aspergillus*-specific IgG at baseline had become sero-negative by week 24 without antifungal treatment. Their x-rays had also improved with a normal picture at week 24.

**Table 2:** Summary of *Aspergillus*-specific antibody titers and total serum IgE in HIV/TB patients in relation to selected baseline characteristics

Test/ Assay/ Cut-off	Factor at TB diagnosis	BASELINE			WEEK 24			Overall P value^$^
		N	Median (IQR)	P value*	N	Median (IQR)	P value*	
Aspergillus IgG ImmunoCAP® cut-off = 40 mg/L	Overall	76	4.4 (2.1,7.2)	NA	94	4.9 (2.1, 9.1)	NA	0.02
	Not on ART	52	4.3 (2.1,7.3)	0.85	65	4.9 (2.7,10.4)	0.35	
	On ART	24	4.8 (2.0,6.9)		28	2.8 (2.0,7.5)		
	CD4 < 100/μL	26	6.2 (2.7,8.7)	0.01	31	5.3 (2.1,12.7)	0.47	
	CD4 ≥ 100/μL	47	3.2 (2.0,6.0)		59	4.8 (2.1,7.5)		
	Coughed for <3 weeks	27	5.2 (2.1,8.5)	0.50	29	4.9 (2.3,10.4)	0.96	
	Coughed for ≥3 weeks	49	4.3 (2.0,7.1)		64	4.9 (2.1,9.0)		
	No Chest pain	41	5.4 (2.7,8.2)	0.03	55	4.8 (2.1,8.8)	0.95	
	Chest pain	35	2.9 (2.0,6.2)		38	5.1 (2.4,10.1)		
	Abnormal Chest X-Ray	59	4.3 (2.0,7.1)	0.79	75	4.9 (2.1,8.8)	0.88	
	Normal Chest X-Ray	6	3.9 (2.6,8.7)		6	3.1 (2.1,10.9)		
Aspergillus IgG Immulite® cut-off = 20 mg/L	Overall	75	7.5 (6.1, 10.3)	NA	87	7.2 (6.1, 9.96)	NA	0.66
	Not on ART	52	8.0 (6.7,10.6)	0.03	61	7.6 (6.2,10.5)	0.27	
	On ART	23	6.6 (5.8,8.2)		26	6.9 (6.1,8.8)		
	CD4 < 100/μL	25	7.9 (5.9,9.1)	0.94	29	7.8 (6.0,11.3)	0.60	
	CD4 ≥ 100/μL	47	7.5 (6.1,10.4)		56	7.0 (6.1,9.5)		
	Coughed for <3 weeks	26	7.3 (6.0,8.8)	0.37	28	7.6 (5.9,10.5)	0.98	
	Coughed for ≥3 weeks	49	7.5 (6.4,10.7)		59	7.2 (6.2,8.8)		
	No Chest pain	40	7.5 (6.4,10.8)	0.54	51	7.5 (6.1,9.7)	0.92	
	Chest pain	35	7.9 (6.0,9,3)		36	7.2 (6.1,10.8)		
	Abnormal Chest X-Ray	58	7.5 (6.1,9.1)	0.30	69	7.0 (6.1,10.1)	0.80	
	Normal Chest X-Ray	6	6.8 (5.0,8.2)		6	8.2 (7.5,8.7)		
Total IgE Immulite® cut-off = 1000 IU/mL	Overall	76	379 (129, 908)	NA	85	251 (64, 794)	NA	<0.01
	Not on ART	52	371 (122,1059)	0.82	59	297 (55,1021)	0.53	
	On ART	24	382 (175,837)		26	231 (77,777)		
	CD4 < 100/μL	26	462 (149,1440)	0.11	29	595 (113,1272)	0.03	
	CD4 ≥ 100/μL	47	355 (114,634)		54	185 (57,532)		
	Coughed for <3 weeks	27	207 (48,489)	0.02	26	213 (39,821)	0.49	
	Coughed for ≥3 weeks	49	493 (172,1200)		59	359 (71,794)		
	No Chest pain	41	355 (149,605)	0.40	49	183 (55,570)	0.06	
	Chest pain	35	489 (121,1221)		36	460 (72,1192)		
	Abnormal Chest X-Ray	59	457 (149,1055)	0.53	68	332 (66,835)	0.54	
	Normal Chest X-Ray	6	401 (322,1487)		6	153 (73,292)		
						NORMAL (≤0.35), n(%)	HIGH (>0.35), n(%)	*P*-VALUE
Aspergillus IgE ImmunoCAP® cut-off = 0.35 kU/L	Overall, *N* = 93	NOT APPLICABLE				84 (90.3)	9 (9.7)	
	Not on ART					59(90.8)	6(9.2)	0.99
	On ART					25(89.3)	3(10.7)	
	CD4 < 100/μL					26 (83.9)	5 (16.1)	0.26
	CD4 ≥ 100/μL					56 (93.3)	4 (6.7)	
	Coughed for <3 weeks					25(86.2)	4(13.8)	0.45
	Coughed for ≥3 weeks					59(92.2)	5(7.8)	
	No Chest pain					51(92.7)	4(7.3)	0.48
	Chest pain					33(86.8)	5(13.2)	
	Abnormal Chest X-Ray					67(89.3)	8(10.7)	0.99
	Normal Chest X-Ray					6(100.0)	0 (0.0)	

Data presented are diagnostic cut-offs, antibody median (interquartile ranges) and *P*-values comparing antibody titers between selected parameters, baseline and end of TB treatment (wk24)

*NA* not applicable

*P-values were obtained using Kruskal-Wallis equality-of-populations rank test. $ *P*-values were obtained using Wilcoxon matched-pairs sign-rank test because of skewed data

Seven patients were negative for all the TB diagnostics (i.e. smear, genexpert, MGIT, LJ) but with abnormal chest x-rays. For these patients, a presumptive diagnosis of TB was made based on the abnormal chest x-ray. However, on comparing the baseline and week 24 x-rays, they all showed marked improvement, but only two had normal week 24 x-rays. These seven patients had *Aspergillus*–specific IgG antibodies in the range of <2 to 7.22 mg/l at baseline and <2 to 21.8 mg/l at week 24. The higher titers at week 24 than baseline could suggest that there is an increase in the aspergillus colonization due to the increasing pulmonary abnormalities especially cavities caused by TB. However, for these seven patients, both titers were below the diagnostic cut-off and not significantly different (*P* > 0.05).

**Fig. 1:**
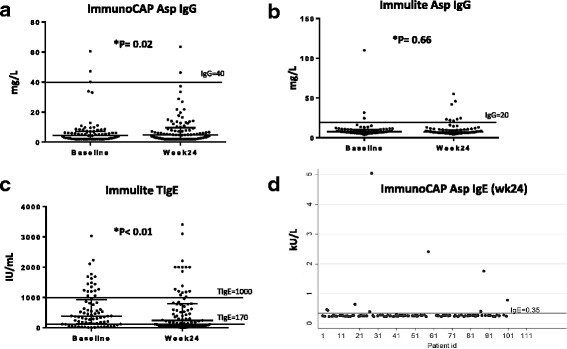
Scatter plots of antibody levels at baseline and week 24. **a** Shows a significant difference in *Aspergillus*-specific IgG levels between baseline and week 24 using ImmunoCAP. **b** Shows no significant difference in *Aspergillus*-specific IgG levels between baseline and week 24 using Immulite. **c** Shows a significant difference in total serum IgE levels between baseline and week 24 using Immulite. **d** Shows distribution of *Aspergillus*-specific IgE levels at week 24

There was a significant difference in the *Aspergillus*-specific IgG antibody levels between baseline and week 24 (*P* = 0.02) (Fig 1a). Patients with a baseline CD4 T-cell count <100 cells/μl had significantly higher median baseline *Aspergillus*-specific IgG titers (*n* = 26, median = 6.2 mg/l, IQR = 2.7 to 8.7) than those with a baseline CD4 T-cell count of ≥100 cells/μl (*n* = 47, median = 3.2 mg/l, IQR = 2.0 to 6.0) (*P* = 0.01). Patients with chest pain at baseline had significantly lower median *Aspergillus*-specific IgG titers (*n* = 35, median = 2.9 mg/l, IQR = 2.0 to 6.2) than those without chest pain (*n* = 41, median = 5.4 mg/l, IQR = 2.7 to 8.2) (*P* = 0.03) (Table 2). Chest pain is not a specific symptom. Out of the 41/101 patients who had chest pain, only one had elevated IgG levels. Out of the 60/101 patients without chest pain, only two had elevated IgG levels. The observed outcome could be explained by other factors related to TB but not CPA.

### Aspergillus-specific IgG antibody levels using Immulite 2000®

Using the Immulite immunoassay, the median *Aspergillus*-specific IgG antibody levels were 7.52 mg/l (IQR, 6.08–10.3) with 4% (3/75) of the patients having elevated titers at baseline; while at end of week 24, the median *Aspergillus*-specific IgG antibody levels were 7.21 mg/l (IQR, 6.08–9.96) with 9.2% (8/87) having elevated levels (Table 2). Only three patients (different from the 3 in ImmunoCAP) were positive at baseline and two of them remained positive at week 24, but with lower antibody titers. Seven patients were negative for all the TB diagnostics (i.e. smear, genexpert, MGIT, LJ) but with abnormal chest x-rays. These seven patients had *aspergillus* IgG antibodies in the range of 4.26 to 8.23 mg/l at baseline and 4.31 to 14.9 mg/l at week 24.

There was no significant difference in the *Aspergillus*-specific IgG antibody levels between baseline and week 24 (*p* = 0.66) (Fig 1b). Patients who were not on antiretroviral therapy at TB diagnosis had significantly higher median *Aspergillus*-specific IgG titers (*n* = 52, median = 8.0 mg/l, IQR = 6.7 to 10.6) than those who were already taking antiretroviral therapy at TB diagnosis (*n* = 23, median = 6.6 mg/l, IQR = 5.8 to 8.2) (*P* = 0.03) (Table 2).

### Total serum IgE using Immulite 2000®

Total serum IgE was measured using Immulite assay with a cut-off of 170 IU/ml. The median total serum IgE levels were 379 IU/ml (IQR, 129–908) with 71% (54/76) of the patients having elevated titers at baseline; while at week 24, the median total serum IgE levels were 251 IU/ml (IQR, 64–794) with 58% (49/85) of patients having elevated levels (Table 2). We then analyzed results using a cut-off to 1000 IU/ml. Only 24% (18/76) of the patients had total serum IgE greater than 1000 IU/mL at baseline and 21% (18/85) at week 24. There was a significant difference in the total serum IgE antibody levels between baseline and week 24 (*p* < 0.01) (Fig 1c).

Patients who had coughed for more than three weeks at TB diagnosis had significantly higher median total serum IgE titers (*n* = 49, median = 493 IU/ml, IQR = 172 to 1200) than those who had coughed for less than three weeks at TB diagnosis (*n* = 27, median = 207 IU/ml, IQR = 48 to 489) (*P* = 0.02). Patients with a CD4 T-cell count <100 cells/μl at week 24, had significantly higher median total serum IgE titers (*n* = 29, median = 595 IU/ml, IQR = 113 to 1272) than those with a baseline CD4 T-cell count of ≥100 cells/μl (*n* = 54, median = 185 IU/ml, IQR = 57 to 532) (*P* = 0.03) (Table 2).

### Aspergillus-specific IgE antibody levels using ImmunoCAP®

*Aspergillus*-specific IgE antibody levels were measured at end of TB treatment only (Fig 1d), and were elevated in 9.7% (9/93) of the patients using a diagnostic cut-off of <0.35kU/l (Table 2). There was no significant relationship between *Aspergillus* sensitization (Asp IgE) and baseline characteristics or respiratory symptoms.

## Discussion

The study demonstrated that *Aspergillus*-specific IgG antibodies were elevated in 4% of HIV-infected Ugandan adults at the start of TB treatment and in 9% at the end of TB treatment. Using ImmunoCAP, participants with CD4 T-cell counts <100 cell/μl had more elevated *Aspergillus*-specific IgG antibodies (*P* = 0.01). The increased level of immunosuppression could have increased their susceptibility to the opportunistic fungal infection. Hyper IgG levels are common in acquired immune deficiency syndrome (AIDS) patients and represent an unrestrained B-cell response in the absence of T-cells [14]. The ImmunoCAP *Aspergillus*-specific IgG antibody titers were higher with more positives at end of TB treatment than baseline, even though the difference in means was small. However, this difference was statistically significant (*P* = 0.02). Initiation of antiretroviral therapy and anti-TB medication could have improved the patients’ ability to mount a good immune response at week 24 than baseline.

Patients who were not on antiretroviral therapy at TB diagnosis (68%) had significantly higher *Aspergillus*-specific IgG titers (*P* = 0.03) using Immulite, and 45% (14/31) of them had CD4 T-cell counts less than 200 cells/μl. Increased level of immunosuppression could still be the contributing factor to this increased susceptibility to the opportunistic fungal infection. There was no significant difference in the Immulite *Aspergillus*-specific IgG antibody levels (*P* = 0.66) between baseline and end of TB treatment.

We observed a discrepancy in the *Aspergillus*-specific IgG antibody levels between the ImmunoCAP and Immulite. Previous studies comparing the ImmunoCAP and Immulite have shown that the values for both tests are highly correlated with antibody levels measured by Immulite having a mean of 3 times higher than when measured by ImmunoCAP. However, this correlation is lost at high ImmunoCA*P* values [15]. This ratio may change based on the specific antibody in question. In our current study, this ratio was approximately 1.6 on average.

About 10% (9/93) of the participants had evidence of *Aspergillus* sensitization at the end of TB treatment. This was an expected outcome in this population since persistence of pulmonary cavities after successful pulmonary TB treatment is very common [16] and these cavities are thought to harbour mould spores leading to fungal colonisation. Besides, ABPA can be misdiagnosed as pulmonary TB, with some similar clinical features [10, 17]. However, ABPA is rarely described outside chronic obstructive pulmonary disease (COPD), asthma and cystic fibrosis. There was no record of these three conditions in our participants, and therefore we argue that *Aspergillus* sensitisation is the explanation for these high IgE results, which could represent a strong T-helper 2 (Th2) response. The association between ABPA and pulmonary TB has been weakly described before mainly in case reports [18–20].

In a recent review, CPA in Uganda was estimated to affect up to 22% of TB patients with cavities and 4% in those without cavities [3]. However, in the current population, pulmonary infiltrates were the most common chest x-ray abnormality both at baseline (91%) and week 24 (41%). Only 5% of our participants had pulmonary cavities on chest x-ray at week 24. This did not change much from the 6% cavities registered at baseline. All the five patients with cavities at week 24 had a good treatment outcome (cured) but 3/5 had been diagnosed with smear negative TB at baseline. This result supports previous work indicating persistence of pulmonary cavities after successful pulmonary TB treatment [16].

At week 24, 95% (95/100) of the chest x-rays showed marked improvement from the baseline chest x-rays and only 52% (52/100) were abnormal. Using ImmunoCAP, all patients with normal and abnormal chest x-rays at week 24 were negative for *Aspergillus*-specific IgG (range: <2 to 9). However, using Immulite, there were five positives among those with normal chest x-ray (median 7 [IQR: 5.9, 9.2]) and three positives among those with abnormal chest x-rays (median 7.6 [IQR: 6.3, 10.3]). We were unable to do computed tomography (CT) scans of the chest, which might have provided better definition of any residual abnormalities.

Total serum IgE is a test for general allergic disease and parasitic infections. It is commonly used together with fungal-allergy diagnostics. Using a cut-off of 170 IU/ml for total serum IgE antibodies, 71% of participants had evidence of allergic disease. This reduced to 58% (49/85) at the end of TB treatment. Of these 49 participants with elevated total serum IgE antibodies at the end of TB treatment, 12% (6/49) had elevated *Aspergillus*-specific IgE antibody titers in the range of 0.41 to 2.4, and *Aspergillus*-specific IgG antibody titers in the range of 6.7 to 55.2.

The implication of the raised total serum IgE levels in this population was not obvious. However, there is evidence that total serum IgE levels tend to be more elevated in non-asthmatic Africans than asthmatic Africans [21]. Most scholars attribute this paradox to parasitic infestations in the African population [21, 22]. This paradox therefore calls for the need to re-evaluate the role of total serum IgE levels in asthma in areas with a high gut parasite prevalence. Recent evidence from genotyping ancestry informative markers indicated that African ancestry is a risk factor for elevated total serum IgE levels in African admixed population [23]. Intestinal helminths are also known to raise total serum IgE levels in HIV patients even without a fully functional CD4 T-cell repertoire [24–26].

Raising the cut-off for total serum IgE to 1000 IU/mL reduced the positivity rate to more than 50%. Only 18 participants had total serum IgE greater than 1000 IU/mL at the end of TB treatment. Of these, 28% (5/18) had elevated *Aspergillus*-specific IgE antibody titers in the range of 0.41 to 2.4, and only two of these (2/5) had elevated *Aspergillus*-specific IgG antibody titers (Immulite). Long term coughing for more than three weeks at baseline was also significantly related to increased total serum IgE titers (*P* = 0.02). It is possible that some participants had both TB and some form of allergy at baseline; which would explain the prolonged cough durations.

53% (51/97) of the patients had negative sputum smears. Smear-negative tuberculosis was registered in 13 to 21% of our participants at baseline. Among smear negative patients (*n* = 51), there were 16 positive genexperts, 20 positive MGIT and 13 positive LJ cultures. Seven patients were negative for all the TB diagnostics (i.e. smear, genexpert, MGIT, LJ) but with abnormal chest x-rays. These seven patients were negative for *Aspergillus* IgG antibodies with very low titers at both baseline and week 24. CPA associated with TB constitutes a significant unrecognized public health problem, which is probably being incorrectly identified as ‘smear-negative tuberculosis’ especially in Africa.

Based on data from India, reduced pulmonary function is associated with *Aspergillus* sensitisation, which unfortunately we were unable to measure. Post-tuberculous sequelae include CPA, bronchiectasis [10] and to this we add *Aspergillus* sensitisation. Reduced pulmonary function persists in patients cured of TB [27], and *Aspergillus* sensitisation could be one of the explanations, through poorly understood mechanisms. The prevalence of pulmonary Aspergillosis in HIV patients is underestimated in Africa because of difficulty in accurate diagnosis. We have only had a few epidemiological studies in Uganda [4, 6, 8]. However, in collaboration with The Global Action Fund for Fungal Infections (GAFFI) and the University of Manchester, efforts are being put in place to train more mycologist and build laboratory capacity in Uganda. We hope that this might solve the problem in the near future.

### Study limitations

The major limitation to the study was that we failed to access control samples for comparison of these antibody titers. This would possibly give more useful information. So we agreed to move on without the controls since the primary goal of this study was not to define diagnostic cut-offs for assays that might subsequently be used in a prevalence study in Uganda. Due to the limited published data on fungal disease epidemiology in sub-Saharan Africa, we found a major challenge in defining which diagnostic cut-offs to use for Aspergillus-specific IgG antibodies in both ImmunoCAP and Immulite. Previously published cut-offs range in 10 to 50 mg/l in similar populations. We were unable to do computed tomography (CT) scans of the chest, which might have provided better definition of any residual abnormalities. Similarly, we did not add other tests like culture since we used stored serum samples. Intestinal helminths are known to raise total serum IgE levels in HIV patients even without a fully functional CD4 T-cell repertoire [24–26]. So the implication of the raised total serum IgE levels in this population was not obvious. We observed a discrepancy in the *Aspergillus*-specific IgG antibody levels between the ImmunoCAP and Immulite similar to what has been described before [15]. Cavities are known to be the major predisposing factor to CPA in TB. However, in our population, cavities were seen in only 5% of the participants.

## Conclusion

In conclusion, *Aspergillus*-specific antibody levels were significantly raised in patients being managed for pulmonary tuberculosis. This colonization and/or sensitization by *Aspergillus* antigens may complicate disease progression and treatment outcomes among TB patients. Detection of *Aspergillus* antibodies is an indispensable tool in the diagnosis and management of the patients with pulmonary aspergillosis. However, the detection of Aspergillus-specific antibodies does not imply that the patient has an active fungal disease; further diagnostic tests would be needed to ascertain the presence of pulmonary aspergillosis. More epidemiological studies are needed to explore and expand the utility of *Aspergillus* antibody testing in resource limited setting.

## Additional file

Additional file 1: Study raw data. File contains a full set of raw data for the tests performed and baseline clinical and demographics characteristics. (XLSX 51 kb)

## Abbreviations

ABPA: Allergic bronchopulmonary aspergillosis
ART: Antiretroviral therapy
CD4: Cluster of differentiation 4
COPD: Chronic obstructive pulmonary disease
CPA: Chronic Pulmonary Aspergillosis
CT: Computerized Tomography
Fig: Fig.
HIV: Human immunodeficiency virus
HR: Isoniazid and Rifampicin
HRZE: Isoniazid, Rifampicin, Pyrazinamide and Ethambutol
IDI: Infectious Diseases Institute
IgE: Immunoglobulin E
IgG: Immunoglobulin G
IQR: Interquartile range
ISID: International Society for Infectious Diseases
LJ: Löwenstein–Jensen
MGIT: Mycobacteria growth indicator tube
SD: Standard deviation, n- sample size
SOUTH: Study on Outcomes related to Tuberculosis and HIV drug concentrations In Uganda
TB: Tuberculosis
Th2: T Helper Type 2 Cell
UK: United Kingdom
WHO: World Health Organisation
